# Point of Care Ultrasound Identification and Aspiration of a Neck Lymph Node

**DOI:** 10.24908/pocus.v9i1.16761

**Published:** 2024-04-22

**Authors:** Andrew Moore, Ali Mrad, Leonard Riley, Sonia M Castillo

**Affiliations:** 1 Division of Pulmonary and Critical Care, The University of Kansas Medical Center Kansas City, KS USA; 2 Division of Pulmonary and Critical Care, Kansas City VA Medical Center Kansas City, KS USA

**Keywords:** POCUS, FNA, Lung Cancer, Supraclavicular Lymph Node

## Abstract

The tissue diagnosis and staging of all types of lung cancer is foundational for prognosis and establishing the optimal treatment plan. In order to appropriately stage lung cancer, the highest stage should be established using the 8^th^ edition TNM criteria, where tumor size (T), nodal involvement (N), and metastasis (M) are all taken into account. Establishing a tissue diagnosis may involve the use of CT guided biopsy, navigational bronchoscopy, endobronchial biopsy, EBUS, percutaneous lymph node biopsy and/or excisional biopsy of supraclavicular nodes. It is recommended to proceed with the method that is considered least invasive and provides the highest staging. We present a case of recurrent lung adenocarcinoma diagnosed with real time ultrasound-guided fine needle aspiration of a neck lymph node.

## Case Presentation 

**Figure 1:**
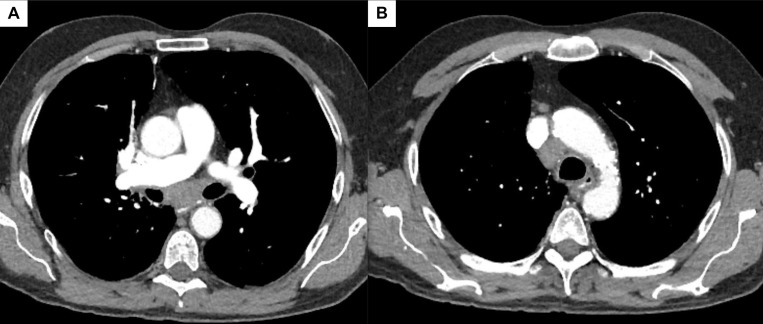
Figure 1: CT scan of the chest with contrast in the mediastinal window and transverse plane showing an enlarged (A) subcarinal and (B) lower right paratracheal lymph node.

A 68-year-old man with a history of orthotopic liver transplant maintained on immunosuppression, right upper lobe adenocarcinoma status post chemotherapy and right upper lobectomy eight years prior, and 45-pack-year history of tobacco disorder presented to the clinic for consultation of his chronic cough. As part of his investigation, he underwent a computed tomography (CT) of the chest that was notable for mediastinal lymphadenopathy in the subcarinal and paratracheal regions (Figure 1A and 1B). There was no reported axillary or supraclavicular adenopathy.

**Figure 2:**
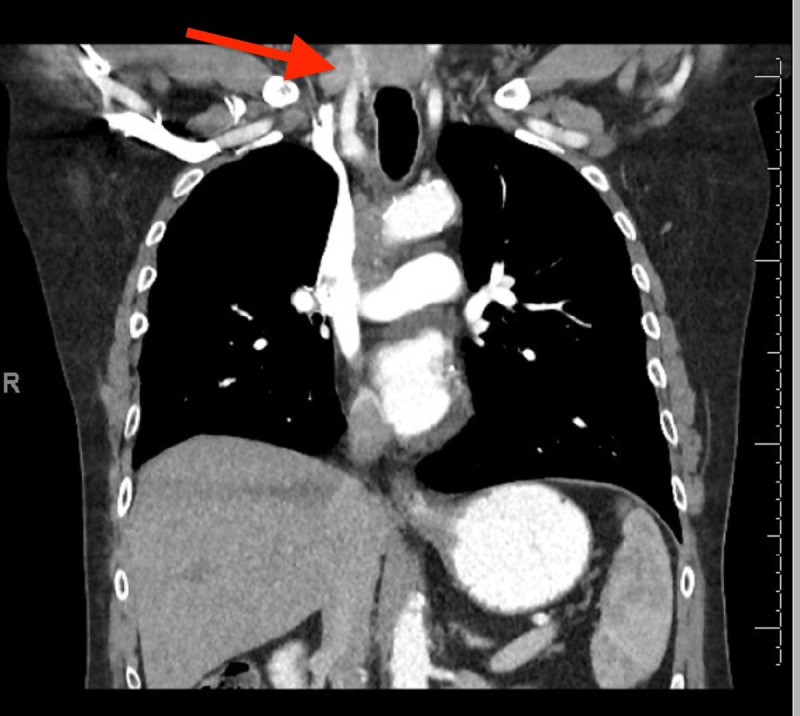
CT scan of the chest with contrast in the mediastinal window and coronal plane showing an enlarged right supraclavicular lymph node.

**Figure 3:**
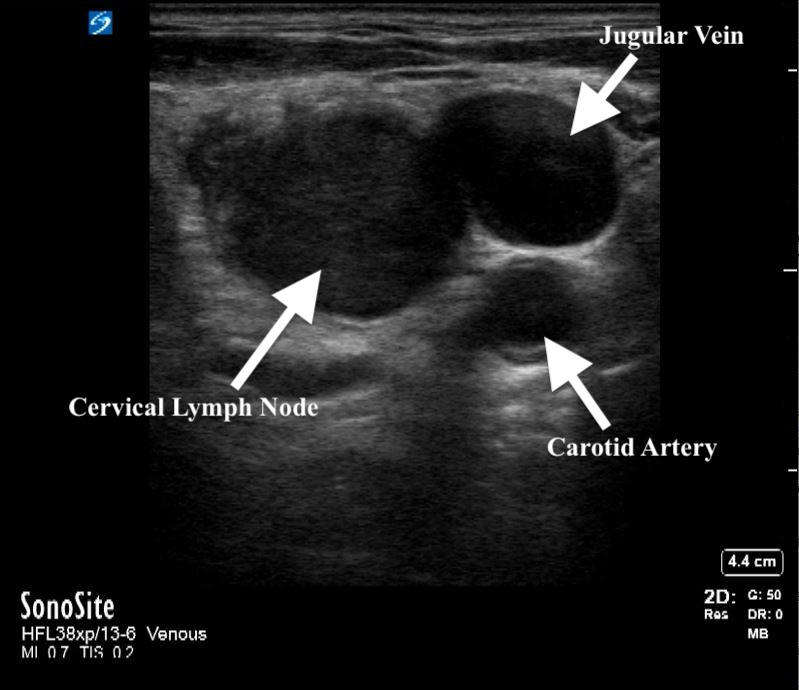
Soft tissue ultrasound image showing an enlarged right supraclavicular lymph node.

He was subsequently referred to pulmonary medicine for endobronchial ultrasound (EBUS) and transbronchial needle aspiration (TBNA) for both diagnostic and staging purposes. However, his scans were reviewed and notable for an enlarged right-sided supraclavicular lymph node, which was not palpable on exam (Figure 2). A point of care ultrasound (POCUS) assessment of his right supraclavicular region with a linear probe demonstrated the findings in Figure 3 and Videos S1 and S2. Based on the patient’s clinical history and findings from the images and videos, we proceeded with ultrasound-guided fine needle aspiration (FNA). This provided a diagnosis of lung cancer and provided staging in a safer and less invasive way than EBUS.

## Discussion

The tissue diagnosis and staging of all types of lung cancer is foundational for prognosis and establishing an optimal treatment plan. In order to appropriately stage lung cancer, the highest stage should be established using the 8th edition TNM criteria where tumor size (T), nodal involvement (N), and metastasis (M) are all taken into account 1 . Current guidelines for non-small cell lung cancer defines N0 disease as no regional lymph node involvement, N1 disease as involvement of ipsilateral peribronchial and/or ipsilateral hilar lymph nodes, N2 disease as involvement of the ipsilateral mediastinal and/or subcarinal lymph nodes, and N3 involvement of any of the following lymph node groups: contralateral mediastinal, contralateral hilar, ipsilateral or contralateral scalene, or supraclavicular nodes 1, 2. In order to establish tissue diagnosis, sampling is needed and can involve the use of CT guided biopsy, navigational bronchoscopy, endobronchial biopsy, EBUS, percutaneous lymph node biopsy, and/or excisional biopsy of supraclavicular nodes. All methods come with their own safety and efficacy profile that include, but are not limited to, pneumothorax, bleeding, infection, bronchospasm, laryngospasm, hypoxemia, hypercarbia, and aspiration 3, 4. These risks are first mediated by proceeding with the method that is considered least invasive and provides the highest staging. 

To evaluate the neck region, CT or ultrasound can be used. When evaluating lymph nodes, ultrasonographic characteristics that are more suggestive of malignancy include larger size (>5 mm), rounded shape (as opposed to oval or reniform), irregular borders, and lack of visible hilum 5. If suspicious nodes are found, further evaluation is needed through either percutaneous needle aspiration or open surgical biopsy. Factors limiting the use of surgical sampling include the need for an incision, bleeding, infection, potential need for sedation and missing the node of interest. All of these factors can be either reduced or eliminated with the use of percutaneous needle aspiration, which has demonstrated its utility in the literature 6, 7. While needle aspiration may have risk of bleeding because of its close proximity to the large neck vessels, it is exceedingly rare when done by a trained provider 8, 9, 10. In a study by El-Shaarawy and colleagues, a neck ultrasound in subjects with evidence of intrathoracic lymphadenopathy found abnormal neck lymph nodes in more than one third of patients 7. Additionally, they performed neck lymph node biopsies in eligible patients, which had a diagnostic yield of 92%, similar to previous reports 6, 7. Importantly, tissue sampling can be carried out by pulmonary physicians and avoids more imaging studies, procedures, and potential adverse effects from sedation and anesthesia 11, 12. 

In our case, we were able to review the suspicious CT and noted the enlarged supraclavicular lymph node that failed to be reported on the formal read. This is a documented blind spot that has been demonstrated by Hassan et al., to miss 18% of cases of abnormally enlarged supraclavicular lymph nodes, with 55% of those being positive for malignancy. A critically important consideration for ensuring proper staging^8^, ^13^. Our initial plan was to pursue EBUS-TBNA to provide tissue sampling and mediastinal staging. However, upon further investigating our patient’s concerning CT findings with POCUS, a suspicious 15 mm right supraclavicular lymph node was found. After discussing the risks, benefits, and alternatives, a percutaneous lymph node biopsy was pursued. The results of our lymph node aspiration were consistent with the patient’s prior adenocarcinoma of the lung. He was referred to oncology and started on systemic chemotherapy. 

**Figure 4:**
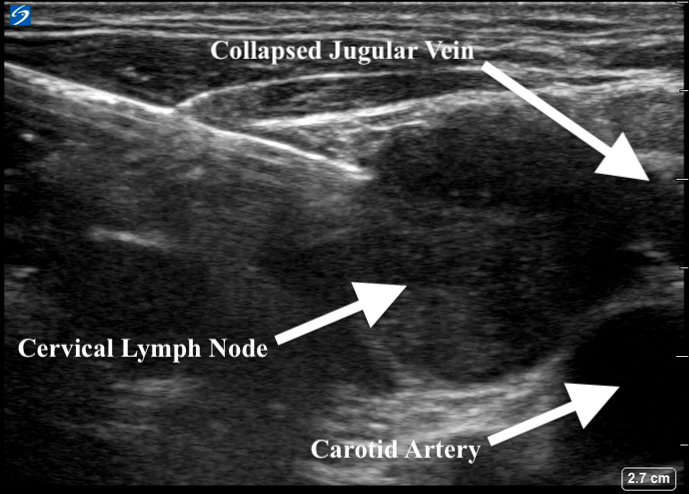
Soft tissue ultrasound image showing an the enlarged right supraclavicular lymph node withthe needle visible in its entire length.

Practically, suspicious neck lymph nodes are identified using a linear transducer. Lymph nodes are characterized as echodense structures surrounded by a clearly defined hyperechoic capsule that are not collapsible, may have a fatty central hilum, and do not show evidence of blood flow on color or spectral Doppler^5^. Once location is confirmed, the site is cleaned, a local anesthetic is applied, and if needed, additional sedation mirroring other routine subcutaneous procedures is provided. In our practice, sampling of the identified lymph is done under real-time ultrasound guidance and an in-plane needle approach with 3-5 passes using an 18, 21, or 22 gauge needle and 10 cc syringe assembled in a needle gun (Figure 4, Video S3). This is akin to a version of the traditional view seen with EBUS-TBNA sampling. Each pass is evaluated with rapid on-site examination by the cytopathology team in the procedure room.

## Supplementary Material

 Video S1 Linear probe with vascular settings evaluating the right supraclavicular region.

Video S2 Linear probe with vascular settings evaluating the right supraclavicular region with color flow Doppler.

Video S3 Linear probe with vascular settings with needle aspiration of the suspicious lymph node.
